# Symptoms of depression in patients with mild cognitive impairment in Parkinson's disease

**DOI:** 10.1590/1980-57642016dn11-020007

**Published:** 2017

**Authors:** Ana Lara Soares Blum Malak, Luiz Felipe Vasconcellos, João Santos Pereira, Denise Vieira Greca, Manuela Cruz, Heloisa Veiga Dias Alves, Mariana Sptiz, Helenice Charchat-Fichman

**Affiliations:** 1 Psychology Department, Pontificial Catholic University of Rio de Janeiro (PUC-Rio), RJ, Brazil; 2 Movement Disorders Sector, Neurology Service, Pedro Ernesto University Hospital, State University of Rio de Janeiro, Rio de Janeiro, RJ, Brazil; 3 Post Graduate Stricto Sensu Program in Medical Sciences, School of Medical Sciences, State University of Rio de Janeiro, Rio de Janeiro, RJ, Brazil

**Keywords:** Parkinson's disease, depression, cognition, mild cognitive impairment, doença de Parkinson, depressão, cognição, declínio cognitivo leve

## Abstract

**Objective:**

To investigate the most frequent depressive symptoms and their association with cognition in Parkinson's disease (PD) patients with mild cognitive impairment (MCI).

**Methods:**

48 patients with PD and 44 controls (CG), aged between 50 and 80 years and with at least 4 years of formal education, all with MCI and none diagnosed with depression, were assessed. Patients and controls were matched for age, education, and Mini-Mental State Examination (MMSE) score. Participants underwent clinical evaluation with a neurologist followed by neuropsychological assessment employing the instruments: MMSE, Clock Drawing Test, Verbal Fluency Test (semantic and phonemic), Figures Memory Test (FMT), Stroop Test, Trail Making Test, Digit Span (WAIS III), Rey Auditory Verbal Learning Test (RAVLT), Hooper Visual Organization Test, and Beck Depression Inventory (BDI).

**Results:**

The most frequent depressive symptoms in the PD group were: difficulty working, fatigue and sleep disorders (the latter also being present in CG). BDI score correlated negatively with learning and recognition memory in both groups. Episodic memory, evaluated by the FMT and RAVLT tests, was the cognitive function showing greatest impairment.

**Conclusion:**

Some of the depressive symptoms observed in PD patients with MCI seem to be attributable to complications of PD, while others are common to both PD and MCI, making differential diagnoses complex but crucial.

## Abstract (pt)

**Objetivo:**

Investigar os sintomas depressivos mais frequentes e sua associação com a cognição de indivíduos com doença de Parkinson (DP) e declínio cognitivo leve (DCL)

**Métodos:**

Foram avaliados 48 pacientes com DP e 44 Controles (GC), com idade entre 50 e 80 anos e escolaridade superior a 4 anos, todos com DCL e sem diagnóstico de depressão. Pacientes e controles foram pareados quanto à idade, escolaridade e pontuação do Mini Exame do Estado Mental (MEEM). Os participantes foram submetidos à avaliação clínica com neurologista seguida de avaliação neuropsicológica com os instrumentos MEEM, Teste do Desenho do Relógio, Teste de Fluência Verbal Semântica e Fonêmica, Teste de Memória de Figuras (FMT), Teste Stroop de Cores e Palavras, Teste das Trilhas (TMT), Span de Dígitos WAIS III, Teste de Aprendizagem Auditivo-Verbal de Rey (RAVLT), Teste de Organização Visual de Hooper e Inventário de Depressão de Beck (BDI).

**Resultados:**

Os sintomas depressivos mais frequentes no grupo DP foram dificuldade para trabalhar, fatigabilidade e distúrbios do sono, sendo este comum ao grupo controle. A pontuação do BDI correlacionou-se negativamente com aprendizagem e memória de reconhecimento em ambos os grupos. Neste estudo, a memória episódica, avaliada pelos testes FMT e RAVLT, apresentou-se como a função cognitiva com maior comprometimento.

**Conclusão:**

Alguns sintomas depressivos prevalentes nos Parkinsonianos com DCL podem ser atribuídos à DP, dificultando o diagnóstico diferencial.

## INTRODUCTION

Parkinson's disease (PD) is one of the most common neurological disorders in the elderly population, accounting for up to two-thirds of patients who seek major movement disorder centers worldwide.^1^ PD has a higher incidence in men than in women and no differences are evident among races and ethnic groups.^2^ The greatest risk factor for PD is age, with the appearance of the first symptoms typically between the fourth and seventh decades of life.

PD is characterized by the occurrence of movement disorders and changes in fine motor control. The clinical signs include resting tremor, bradykinesia, muscular rigidity, lack of voluntary movements and changes in balance.^3^ These signs result from the loss of dopaminergic neurons in a region of the midbrain called the substantia nigra pars compacta.

Unlike the motor symptoms, there are also nonmotor manifestations of the disease. Psychosis, cognitive impairment, sleep disorders, depression, among others, are common symptoms and should not be ignored, since they entail substantial losses in patient quality of life.^4^ However, studies show that 62% of nonmotor symptoms such as pain, apathy, sexual disorders and constipation are not reported, as patients do not recognize such symptoms as belonging to PD.^5^ The few reports and failure to recognize these symptoms have important social and therapeutic implications. Left untreated, these symptoms can have a major impact on the lives of these patients and are often the leading cause of hospitalization and institutionalization.^6^

Describing the disease in 1817, James Parkinson postulated that the intellect would be preserved even in advanced stages. For many years, PD had been described as a disease with strictly motor disturbances, which contributed to the neglecting of the cognitive and psychiatric dysfunctions associated with the illness.^7^ In the last two decades, however, there has been a growing interest in the cognitive changes in PD, favoring the idea that the clinical spectrum of this disease is wider than initially considered.^6^ Studies have shown the presence of cognitive decline even in early stages of the disease, with 70% of patients without apparent cognitive decline showing impairments when formally assessed^8,9^ In these cases, cognitive decline is characterized by a very evident executive dysfunction, commonly associated with apathy and bradyphrenia, combined with relative preservation of memory, language and praxis.^9^

The validity of PD-MCI as a clinical entity is supported by converging functional, morphological neuroimaging, electroencephalography, neuropsychological, genetic and histological data showing an association between neuropathophysiological variables and cognitive impairment in PD patients.^10^ The Movement Disorders Society Task Force (MDS) criteria for PD-MCI indicates a two-step process: level I and level II. In level I, MCI is diagnosed when there is impairment on a cognitive global scale or on at least two neuropsychological tests (abbreviated assessment); and level II with a comprehensive neuropsychological assessment, requiring at least one compromised domain (on at least two neuropsychological tests) or two compromised domains (on one neuropsychological test), allowing the evaluation of MCI subtypes.^17^

Among the non-motor symptoms, depression is the most commonly associated with PD, affecting 40% of patients.^11^ The etiological factors of depression in PD include an imbalance of neurotransmitters and also functional loss due to the progression of this disease. Remy et al.^12^ believe that dopamine is also linked to non-motor aspects of PD. According to these authors, a dysfunction in the limbic system combining dopamine, serotonin, and norepinephrine is involved in triggering depression. Uemura et al.^13^ stated that subjects with PD are twice as likely to develop depression compared to individuals who do not have the disease. The authors correlated depressive symptoms with cognitive impairment and motor disorders, suggesting that endogenous changes in the neurotransmitter system, besides the natural progression of the disease, contributed to the onset of depressive symptoms. Similarly, Chagas et al.^14^ investigated the association between depression and PD, concluding that this association is stronger for those with cognitive deficits. The comorbidity between PD and depression becomes greater with increasing cognitive impairment, suggesting a two-way mechanism, where depression and cognition are mutually influenced.

The prevalence of depressive symptoms in PD is highly variable, which can be explained by type of population studied, instruments used for the diagnosis of depression, type of depression investigated and the statistical methods used in the studies. Thus, studies adopting rating scales to quantify depressive symptoms tend to have higher prevalence rates than those using manuals to define diagnoses (Diagnostic and Statistical Manual of Mental Disorders-DSM, International Classification of Diseases-ICD, American Psychological Association-APA). Studies using self-assessment scales have reported higher prevalence rates of depression than studies with scales filled out by family members or caregivers. The lowest prevalence rates are found in population studies, unlike those conducted with institutionalized subjects or users of outpatient services.^15^

Few studies have explored the effects of depressive symptoms on cognition in PD patients. The overlap of these symptoms with those characteristic of PD makes it difficult to differentiate which are inherent to one or the other. There is now consensus on the idea that nonmotor aspects of PD, such as cognitive and psychiatric disorders, contribute to the progression of the disease. Therefore, they are linked to complications such as loss of autonomy and functionality, decreased quality of life for both patients and caregivers and, finally, high institutionalization and mortality rates. In this context, the present study aimed to investigate the impact of depressive symptoms on the cognition of PD patients, comparing the profile of these symptoms in PD patients and controls, as well as determining which cognitive measures are affected by the presence of depression symptoms. This study is justified by the need to further our current understanding regarding the influence of depressive symptoms on PD with MCI, describing what is typical for each pathology in order to enable better clinical and treatment planning strategies.

## METHODS

The study was conducted between January 2014 and September 2015. It is part of a larger project that aims to identify predictors of dementia in PD, conducted by the Neurology Service, Pedro Ernesto University Hospital, State University of Rio de Janeiro, RJ, Brazil in collaboration with the clinical neuropsychology research group of Pontificial University Catholic of Rio de Janeiro (PUC-Rio), RJ, Brazil.

**Participants.** A total of 132 subjects, with ages ranging between 50 and 80 years, with at least 4 years of formal education, were evaluated. Initially there were two groups: PD and CG. None of the subjects in either group had diagnostic criteria for DSM-IV depression but all had a diagnosis of MCI.

In the PD group, 8 subjects were excluded due to MMSE scores below the established cutoff point;^16^ 7 subjects for not fulfilling the criteria for MCI^17^ and 3 subjects for having a diagnosis of depression according to DSM-IV criteria. Therefore, 48 subjects comprised the PD group. All subjects fulfilled the criteria for PD in accordance with the United Kingdom Parkinson's Disease Society Brain Bank (UKPDSBB), having time with disease of greater than three but less than ten years, and scores of between 2 and 4 on the Hoehn-Yahr scale.

In the CG, 2 subjects were excluded due to MMSE scores,^16^ 17 subjects for not fulfilling the criteria for MCI,^17^ 1 subject for having a diagnosis of depression according to DSM-IV criteria, and 2 subjects due to other clinical issues. Individuals with psychiatric or neurological disorders and current or previous history of alcohol abuse and/or illegal drugs use were excluded. Therefore, 44 subjects comprised the CG.

Subjects from the PD and CG were matched for age, education, and MMSE score.

**Instruments.** Cognitive assessment was performed using a previously assembled neuropsychological testing protocol, comprising the following instruments:

*Mini-Mental State Examination (MMSE)* - screening test designed to quickly measure global cognitive functioning, temporal and spatial orientation, attention, immediate and short-term memory, language, praxis, and calculation.^16^

*Clock Drawing Test (CDT)* - cognitive screening tool that checks praxis and visuoconstructional ability.^18^

*Semantic and Phonemic Verbal Fluency Test* - assesses language, sustained attention, organization, strategy, and perseveration.^19^

*Figures Memory Test (FMT)* - evaluates episodic memory and consists of different tasks: naming and perception, incidental memory (IM), immediate memory (M1), learning (M2), delayed recall (M5), and recognition.^20,21^

*Stroop Color and Word Test* - Victoria version (Stroop) - evaluates selective attention, cognitive flexibility and inhibitory control through the response to specific stimuli while inhibiting more automated processes.^22^

*Trail Making Test (TMT)* - evaluates processing speed, divided attention, and cognitive flexibility.^23^

*Digit Span WAIS III (Digits)* - measures verbal attention, short-term memory and working memory.^24^

*Rey Auditory Verbal Learning Test (RAVLT)* - Evaluates episodic memory, learning, susceptibility to interference (by presenting a list of distracting words), information retention, and recognition.^25^

*Hooper Visual Organization Test (Hooper VOT)* - assesses visuospatial abilities.^26^

*Beck Depression Inventory (BDI)* - evaluates the presence of depressive symptoms and their intensity.^27^

**Procedure.** Initially, patients and controls were submitted to a clinical evaluation with a neurologist specialized in Movement Disorders, where demographic data and information regarding the existence of comorbidities (such as hypertension, diabetes, hyperuricemia, and alcoholism) were collected. The Hohen-Yahr, Unified Parkinson's Disease Rating Scale (UPDRS) and the Parkinson's Disease Questionnaire (PDQ-39) scales were applied, and PD subtype was classified (tremulous, rigid-akinetic or mixed).

Next, the neuropsychological assessment was conducted by trained psychologists, in a single session that lasted for one hour. The assessments were conducted in rooms with adequate testing conditions. All participants were evaluated through the same neuropsychological protocol in the following fixed order: MMSE, FMT, CDT, Semantic Verbal Fluency Test (animals), FMT (delayed recall and recognition), RAVLT, Digit Span (forward and backward), Stroop, TMT (A and B forms), RAVLT (delayed recall and recognition), Phonemic Verbal Fluency Test (FAS), Hooper, and the BDI.

The symptoms of depression were evaluated with the BDI scale in both groups. Based on this, the sample was divided into four groups: patients without depressive symptoms (PD), patients with depressive symptoms (PDDS), controls without depressive symptoms (CG) and controls with depressive symptoms (CGDS).

Some cognitive functions can worsen in the off phase. Thus, all PD patients (with or without depressive symptoms) were evaluated during the ON phase of the medication.

MCI diagnosis, for all PD patients, was performed according to MDS task force criteria level 2, which has higher specificity and requires neuropsychological testing of multiple domains.^17^ For the CG, MCI diagnosis was defined as a clinical condition with a decline in one or more cognitive domains, incongruent with age and schooling, which can affect the ability to perform more complex activities, while maintaining activities of daily living.^28^

Each participant signed an Informed Consent Form approved by the Ethics Committee of Pedro Ernesto University Hospital, placed on Plataforma Brasil (number 486.273).

**Statistical analyses.** The analyses were performed using the Statistical Package for Social Sciences (SPSS) software, version 22. Descriptive analysis was conducted for the frequencies of the following variables: gender, age, education, cognition, and depressive symptoms. Student's t-test was used to compare groups (PD and CG) in terms of age, education, MMSE and BDI scores. A Chi-square analysis compared the frequency of depressive symptoms in the two groups. Pearson correlation was conducted between BDI total score and scores on the cognitive tests. In order to compare the neuropsychological performance of patients and controls with and without depressive symptoms, one-way ANOVA was performed, followed by the Bonferroni post hoc test. Statistical significance was set at p<0.05 for all analyses.

## RESULTS

There were no significant differences among groups (PD × PDDS × CG x CGDS) for any of the variables (Age: p=0.170; Education: p=0.414; MMSE: p=0.634), as shown in Table 1.

**Table 1:** Sample characteristics (raw scores).

Variables		Patients × (sd)			Controls × (sd)		p^(2)^
		**PD (n=33)**	**PDDS^(3)^ (n=15)**		**CG (n=28)**	**CGDS^(1)^(n=16)**	
Age (years)		63.0 (7.24)	59.1 (7.12)		60.0 (5.55)	62.0 (8.13)	0.198^(3)^
Education level (years)		10.1 (4.22)	10,1 (3.40)		11.4 (4.48)	11.7 (4.06)	0,430^(3)^
MMSE		27.4 (1.68)	27.4 (2.59)		27.8 (1.23)	27.3 (1.20)	0.634^(3)^
BDI		4.3 (2.97)	14.9 (5.47)		4.8 (2.50)	17.7 (7.81)	< 0.001^(3)^
Sex % (male/female)		69.7 (30.3)	66.7 (33.3)		35.7 (64.3)	25.0 (75.0)	-
Duration of symptoms (months)		72.3 (43.41)	76.80 (39.23)		-	-	0.734
L-dopa equivalent dose (mg)		722.5 (320.32)	730.5 (356.07)		-	-	0.939
Hoehn & Yahr scale		2.09 (0.27)	2.07 (0.18)		-	-	0.748
Schwab-England scale		84.9 (7.12)	83.3 (6.17)		-	-	0.481
UPDRS-I		2.9 (5.49)	2.3 (1.29)		-	-	0.707
UPDRS-II		18.7 (27.50)	11.7 (4.62)		-	-	0.331
UPDRS-III		34.9 (56.79)	19.1 (7.58)		-	-	0.291
UPDRS-IV		2.0 (3.60)	2.2 (2.83)		-	-	0.828
UPDRS total		31.8 (9.08)	35.3 (10.95)		-	-	0.246
PDQ-39 mobility		22.4 (19.75)	42.8 (23.70)		-	-	0.003
PDQ-39 ADL		22.4 (18.84)	35.8 (21.16)		-	-	0.034
PDQ-39 emotional well-being		22.8 (15.20)	46.7 (18.58)		-	-	<0.001
PDQ-39 stigma		15.3 (16.47)	32.9 (29.64)		-	-	0.011
PDQ-39 social support		10.9 (16.47)	22.8 (15.90)		-	-	0.023
PDQ-39 cognition		21.09 (17.45)	33.7 (12.67)		-	-	0.016
PDQ-39 communication		19.0 (16.43)	27.8 (18.54)		-	-	0.108
PDQ-39 bodily discomfort		40.6 (17.55)	56.7 (19.97)		-	-	0.008
PDQ-39 total		21.9 (11.84)	38.7 (14.69)		-	-	< 0.001
PD subtypes (%)	Rigid-akinetic	48.5	53.3		-	-	-
	Mixed	42.4	40.0		-	-	-
	Tremulous	9.1	6.7		-	-	-
MCI subtypes (%)	Amnestic single-domain	6.1	0.0		7.1	6.3	-
	Amnestic multiple-domains	36.4	86.8		50.1	68.7	-
	Non-amnestic single-domain	18.2	6.7		7.1	6.3	-
	Non-amnestic multiple-domains	39.3	6.7		35.7	18.7	-

(1) BDI > 10 points;

(2) Student's t-test;

(3) ANOVA.

**Figure 1:**
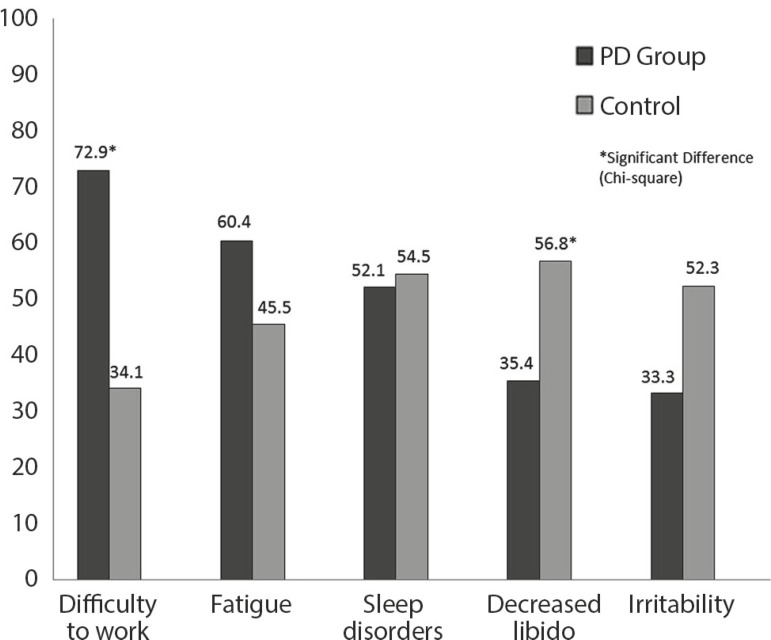
Frequency of depressive symptoms.

For the characterization of depressive symptoms in PD, the most common symptoms in patients and controls were analyzed, as shown in Figure 1. Chi-square analysis indicated a significant difference in the frequencies of the symptoms "decreased libido" and "difficulty working". There was no significant difference for "irritability", "fatigue" and "sleep disorders". Chi-square analysis showed an association between the depressive symptoms "difficulty working" and "fatigue" with the UPDRS (section 1: emotional state) and PDQ-39 (cognition section) scales, respectively. Pearson's correlation analysis showed that, in the PD group, BDI scores correlated negatively with learning (M2: p=0.044; r= -0.292) and with recognition in the RAVLT (p=0.026; r= -0.320). For the control group, BDI score also correlated negatively with learning (M2: p=0.047; r= -0.301), recognition in the RAVLT (p=0.011; r= -0.378), and delayed recall (M5: p=<0.001; r= -0.520 and A7: p=0.029; r= -0.330).

Analysis of variance (ANOVA) was performed, followed by the Bonferroni post hoc test. The performances of the groups on the neuropsychological tests are given in Table 2.

**Table 2:** Performance of patients and controls on the neuropsychological tests (raw scores).

	Neuropsychological tests	Patients × (sd)			Controls × (sd)		F^(14)^	p^(14)^
		**PD (n=33)**	**PDDS^(13)^ (n=15)**		**CG (n=28)**	**CGDS^(13)^(n=16)**		
FMT	Naming	10.0 (0.00)	10.0 (0.00)		9.6 (1.89)	10.0 (0.00)	0.912	0.439
	IM^(1)^	5.3 (1.43)	5.1 (1.51)		5.6 (1.13)	5.5 (0.89)	0.650	0.585
	M1^(2)^	7.5 (1.37)^(a)^	7.4 (0.91)		8.4 (1.20)	8.1 (1.30)	3.401	0.021
	M2^(3)^	8.7 (0.98)	8.5 (1.13)		9.0 (1.39)	8.6 (0.96)	0.912	0.438
	M5^(4)^	7.4 (1.97)^(a)^	7.0 (1.51)^(b)^		8.71 (1.08)	7.9 (1.46)	5.037	0.003
	Recognition	10.0 (0.17)	9.7 (0.46)^(b;c)^		10.0 (0.00)	10.0 (0.25)	4.655	0.005
RAVLT	A1^(5)^	4.5 (1.28)	4.1 (0.83)		5.2 (1.71)	4.9 (1.61)	2.381	0.075
	B1^(6)^	4.8 (1.55)	3.7 (1.40)		4.8 (2.11)	4.5 (2.37)	1.486	0.224
	A6^(7)^	5.8 (3.25)	5.0 (3.03)^(b)^		7.8 (2.91)	6.8 (2.77)	3.486	0.019
	A7^(8)^	6.3 (3.43)	5.0 (2.93)^(b)^		8.5 (3.18)	6.4 (3.05)	4.508	0.005
	LOT^(9)^	15.9 (8.16)	13.9 (5.71)		17.2 (5.95)	15.4 (4.41)	0.847	0.472
	PI^(10)^	1.3 (0.95)	0.9 (0.46)		1.0 (0.47)	1.0 (0.52)	1.606	0.194
	RI^(11)^	0.6 (0.26)^(a)^	0.6 (0.29)		0.8 (0.22)	0.7 (0.25)	3.592	0.017
	FS^(12)^	1.1 (0.72)	1.4 (1.85)		1.1 (0.28)	1.1 (0.47)	0.412	0.744
	Recognition	7.9 (3.97)	4.5 (5.43)		8.4 (4.32)	4.1 (5.44)^(d;e)^	4.924	0.003
Digits	Forward	6.9 (2.53)	7.5 (2.03)		6.2 (1.36)	6.1 (1.18)	2.016	0.117
	Backward	4.7 (1.94)	4.9 (1.41)		4.1 (1.57)	3.8 (1.57)	1.858	0.143
Stroop	Time Card 1 (sec)	19.9 (7.24)	21.2 (14.41)		17.1 (3.59)	20.2 (6.32)	1.116	0.347
	Errors Card 1	0.0 (0.00)	0.1 (0.26)		0.0 (0.19)	0.2 (0.75)	1.095	0.356
	Time Card 2 (sec)	24.6 (7.54)	23.3 (9.85)		20.9 (4.44)	24.8 (8.60)	1.496	0.221
	Errors Card 2	0.0 (0.18)	0.1 (0.52)		0.0 (0.20)	0.0 (0.00)	0.783	0.506
	Time Card 3 (sec)	41.5 (17.82)	41.3 (24.77)		35.2 (11.27)	42.1 (15.05)	0.914	0.438
	Errors Card 3	1.5 (2.00)	2.1 (4.80)		0.8 (2.00)	2.3 (3.57)	1.085	0.360
	Interference effect	2.2 (0.82)	2.1 (0.78)		2.1 (0.66)	2.0 (0.58)	0.283	0.838
TMT	Time A	66.3 (23.17)	65.8 (22.37)		56.0 (22.88)	54.8 (15.84)	1.732	0.167
	Time B	197.3 (122.43)	191.8 (72.03)		156.4 (84.51)	168.2 (91.63)	0.935	0.428
Verbal fluency	Semantic (Animals)	17.3 (3.45)	16.1 (5.0)		17.2 (4.3)	15.7 (3.7)	0.814	0.489
	Phonemic (FAS)	31.3 (9.22)	28.6 (5.41)		32.8 (7.29)	30.3 (9.30)	0.921	0.434
Clock Drawing Test		7.2 (2.41)	7.4 (2.26)		7.3 (1.88)	7.0 (2.07)	0.104	0.958
Hooper VOT		16.8 (4.92)	17.1 (4.79)		18.1 (4.90)	18.4 (4.45)	0.596	0.619

(1) IM: incidental memory.

(2) M1: number of figures recalled after 1-minute visualization.

(3) M2: learning.

(4) M5: recall of memorized figures after 5 minutes.

(5) A1: retrieval of words present in a list of 15 nouns.

(6) B1: retrieval of words included in the interference list of 15 new nouns.

(7) A6: number of words memorized from list A without reread.

(8) A7: number of words memorized from list A, without reread, after 20-minute interval.

(9) LOT: learning over trials.

(10) PI: Proactive Interference (B1/A1).

(11) RI: Retroactive Interference (A6/A5).

(12) FS: forgetting speed (A7/A6).

(13) BDI > 10 points.

(14) ANOVA.

(a) Significant difference between CG and PD.

(b) Significant difference between CG and PDD.

(c) Significant difference between PD and PDD.

(d) Significant difference between PD and CGD.

(e) Significant difference between CG and CGD.

## DISCUSSION

The aim of the present study was to investigate the impact of depressive symptoms on the cognition of PD patients with MCI, correlating the severity of these symptoms with different cognitive measures, and comparing them to a control group.

The literature points to a difficulty in diagnosing depression in PD because symptoms overlap with those of the disease itself.^29,30^ In this study, the most frequent depressive symptoms in the PD group were difficulty working, fatigue, and sleep disorders, which might be ascribed exclusively to PD. This result is supported by the low frequency of these symptoms in the CG, except for sleep disorders, which was present in both groups. The CG presented, more often, irritability symptoms and decreased libido, which is characteristic of depression. This difference in the profile of depressive symptoms between the groups confirms the hypothesis that, in PD, they may be secondary to disease symptoms and are probably not due to depression. Cimino, Siders & Zesiewicz^31^ associated the symptoms of depression to PD's rapid progression, impairment in activities of daily living and the most severe cognitive impairments. According to these authors, the depressive symptoms cause greater impact on the functionality of a PD patient than the motor difficulties arising from the disease.

The results showed a negative correlation between BDI and learning (FMT), and also between BDI and recognition memory (RAVLT), both in the PD group and in the CG. Thus, more depressive symptoms, regardless of PD, caused worse performance on these tests. Sleep disorders were present in both groups, suggesting an association between this symptom and worse performance. Studies have shown that sleep is involved in learning and memory process consolidation and concluded that sleep disorders in PD, besides reducing quality of life, cause cognitive deficits in memory, learning and executive functions.^32^ Sleep disorders are more prevalent in subjects with MCI than in those without cognitive impairment, according to a review by Silva.^33^

In the CG, BDI scores correlated negatively with delayed episodic memory (FMT and RAVLT), showing an association between the presence of depressive symptoms and poor performance on these tasks. Impaired episodic memory is a predictive factor of conversion to dementia, particularly in free recall tasks after a time interval.^34^ Avilla & Bottino^35^ reported that verbal and delayed recall memory tests are effective tools for differentiating patients with dementia from those with depression, as they, despite having an impaired learning capacity, can store information and recall it after a time interval. Therefore, changes in this specific form of memory may serve as an indication of future dementia. Modrego & Ferrández^36^ concluded that combined depression and MCI doubles the risk of conversion to Alzheimer's disease (AD) compared to individuals without depression.

Regarding performance on the neuropsychological assessment, the PD and control groups showed similar results on most tests that made up the battery. Statistically significant differences were observed only in the FMT and RAVLT (Table 2). The PD group had worse performance on late anterograde episodic memory (FMT) and retroactive interference (RAVLT). The results suggest that these cognitive impairments are related to PD with MCI, regardless of the presence of depressive symptoms. Although the literature indicates frontal executive deficits as prevalent in PD, some authors have concluded that MCI in this disease cannot be characterized only by this type of impairment. Muslimovic et al.^37^ confirmed this hypothesis by demonstrating the existence of cognitive impairments in early stages of the disease, not only in executive functions, but also in memory. More than 50% of PD patients have some type of cognitive impairment, 20% predominantly related to memory deficits, 30% to executive dysfunctions, and 50% to decline in global cognitive function.^38^ However, studies show that PD patients exhibit no decline in recognition memory.^7,39^ The impaired recognition performance in the PDDS group, when compared to the PD group, suggests an influence of the association between depressive symptoms and MCI, given the same task showed a decline when the performance between the CGDS and PD groups, and the CGDS and CG groups were compared. Therefore, PDDS and CGDS had impaired recognition performance compared to the groups without depressive symptoms. These findings are in line with the study of Nebes et al.^40^ which concluded that access to cognitive resources becomes deficient in depressed subjects, leading to cognitive decline on several neuropsychological tasks, especially episodic memory. They ratify the hypothesis that the combination of depression and MCI significantly increases the risk of conversion to AD,^36^ since impairment in recognition memory is a predictive factor for the development of this type of dementia.

The results of the present study indicated changes in episodic memory and attention in the PDDS group when compared to the CG, corroborating the notion that depressive symptoms influence the extent, but not severity, of cognitive deficits in PD patients.^11^ The PDDS and the CGDS groups demonstrated a similar pattern of performance on the neuropsychological tests. Both had worse results compared to the PD and CG groups, in accordance with the literature, which states that depression worsens cognition regardless of the existence of another pathology.

This study has the limitation of not including a PD group with MCI diagnosed with depression according to DSM-IV criteria. The fact that these patients only had symptoms and not a diagnosis of depression, probably contributed to the fact that both groups had similar performance on most of neuropsychological tests employed.

It is known that in PD, both MCI and depression are risk factors for dementia, making early detection and treatment of these two conditions vital.^36^ Biundo et al.^41^ reported that each year, between 9 and 15% of PD patients evolve to dementia. In addition, depression and MCI directly affect autonomy and independence, resulting in poor quality of life and high rates of institutionalization and mortality. In many cases, depressive symptoms can mimic MCI or preclinical dementia, making the differential diagnosis and treatment of these pathologies crucial.^35^ Trambley et al.^42^ emphasized the heterogeneity of cognitive impairment in PD. They reported that many researchers characterize PD patients into subtypes according to different criteria, such as predominant motor symptom, age of onset of the disease, and the presence of depression, which is one of the most commonly used criterion to categorize them cognitively. Thus, the importance of early detection of these symptoms is clear, characterizing MCI in PD, as this condition increases the likelihood of conversion to dementia.

In summary, the most frequent depressive symptoms in the PD group were difficulty working, fatigue, and sleep disorders. Furthermore, episodic memory and attention were the cognitive domains most impacted by depressive symptoms. Understanding the specific profiles in PD and MCI, with and without depression, may facilitate appropriate care of these patients. In this context, future studies should include patients with PD and MCI and depression, enabling characterization of their neuropsychological profile.
